# Post-Translational Inhibition of IP-10 Secretion in IEC by Probiotic Bacteria: Impact on Chronic Inflammation

**DOI:** 10.1371/journal.pone.0004365

**Published:** 2009-02-06

**Authors:** Gabriele Hörmannsperger, Thomas Clavel, Micha Hoffmann, Caroline Reiff, Denise Kelly, Gunnar Loh, Michael Blaut, Gabriele Hölzlwimmer, Melanie Laschinger, Dirk Haller

**Affiliations:** 1 Chair for Biofunctionality, ZIEL-Research Center for Nutrition and Food Science, Technische Universität München, Freising-Weihenstephan, Germany; 2 Rowett Institute of Nutrition and Health, Aberdeen University, Aberdeen, United Kingdom; 3 Gastrointestinale Mikrobiologie, Deutsches Institut für Ernährungsforschung, Potsdam-Rehbrücke, Nuthetal, Germany; 4 Helmholz-Zentrum München, Institute for Pathology, München-Neuherberg, Germany; 5 Department of Surgery, Technische Universität München, Munich, Germany; East Carolina University, United States of America

## Abstract

**Background:**

Clinical and experimental studies suggest that the probiotic mixture VSL#3 has protective activities in the context of inflammatory bowel disease (IBD). The aim of the study was to reveal bacterial strain-specific molecular mechanisms underlying the anti-inflammatory potential of VSL#3 in intestinal epithelial cells (IEC).

**Methodology/Principal Findings:**

VSL#3 inhibited TNF-induced secretion of the T-cell chemokine interferon-inducible protein (IP-10) in Mode-K cells. *Lactobacillus casei* (*L. casei*) cell surface proteins were identified as active anti-inflammatory components of VSL#3. Interestingly, *L. casei* failed to block TNF-induced IP-10 promoter activity or IP-10 gene transcription at the mRNA expression level but completely inhibited IP-10 protein secretion as well as IP-10-mediated T-cell transmigration. Kinetic studies, pulse-chase experiments and the use of a pharmacological inhibitor for the export machinery (brefeldin A) showed that *L. casei* did not impair initial IP-10 production but decreased intracellular IP-10 protein stability as a result of blocked IP-10 secretion. Although *L. casei* induced IP-10 ubiquitination, the inhibition of proteasomal or lysosomal degradation did not prevent the loss of intracellular IP-10. Most important for the mechanistic understanding, the inhibition of vesicular trafficking by 3-methyladenine (3-MA) inhibited IP-10 but not IL-6 expression, mimicking the inhibitory effects of *L. casei*. These findings suggest that *L. casei* impairs vesicular pathways important for the secretion of IP-10, followed by subsequent degradation of the proinflammatory chemokine. Feeding studies in TNF^ΔARE^ and IL-10^−/−^ mice revealed a compartimentalized protection of VSL#3 on the development of cecal but not on ileal or colonic inflammation. Consistent with reduced tissue pathology in IL-10^−/−^ mice, IP-10 protein expression was reduced in primary epithelial cells.

**Conclusions/Significance:**

We demonstrate segment specific effects of probiotic intervention that correlate with reduced IP-10 protein expression in the native epithelium. Furthermore, we revealed post-translational degradation of IP-10 protein in IEC to be the molecular mechanism underlying the anti-inflammatory effect.

## Introduction

Inflammatory bowel diseases (IBD) are spontanously relapsing, immunologically mediated disorders of the gastrointestinal tract. The incidence and prevalence of the two main idiopathic pathologies of IBD, Crohn's disease (CD) and ulcerative colitis (UC), are rising globally in parallel to the progression of the industrialisation. More that 3.5 million people suffer from IBD in the United States and Europe [1], but although extensive research was performed in this field, the initial trigger is still unknown and efficient therapies are not available yet. Genetical susceptibility of the host [2] and the intestinal microbiota both were found to play a pivotal role in the onset and perpetuation of IBD. Enhanced numbers of mucosa associated bacteria and decreased microbiota diversity are associated with these diseases [3]. In contrast to the fact, that IBD is the result of an overreaction of the intestinal immune system towards intestinal microbes, clinical studies showed that oral uptake of specific probiotic bacterial strains like VSL#3 [4], [5] or *Escherichia coli Nissle* 1917 [6], [7] resulted in attenuation of IBD disease severity [8]. VSL#3, a mixture of eight different lactic acid bacteria (*Lactobacillus* (*L*.) *acidophilus*, *L. bulgaricus*, *L. casei, L. plantarum, Streptococcus thermophilus*, *Bifidobacterium* (*B*.) *breve*, *B. infantis*, *B. longum*) was effective in the prevention and in the maintenance treatment of pouchitis and UC [9]–[14]. Apart from its protective effect in clinical studies, VSL#3 was shown to reduce experimental colitis in IL-10-deficient (IL-10^−/−^) mice. Treatment of IL-10^−/−^ mice resulted in decreased histopathology, normalization of colonic function, barrier integrity and mucosal cytokine production [15]. Furthermore, VSL#3 was shown to induce protective heat-shock-proteins in intestinal epithelial cells (IEC) [16] or proliferation of IL-10-dependent TGFβ-bearing regulatory T-cells in Th1-dependent murine colitis [17]. However, there is no study showing protective effects of VSL#3 in the context of CD or experimental ileitis, suggesting disease-and intestinal segment-specific effects of VSL#3. Extensive progress has been made in understanding probiotic effects of VSL#3 in the context of IBD but the molecular mechanisms as well as strain-specificity remain to be elucidated.

IEC are crucial for maintaining intestinal homeostasis [18] and failure to control inflammatory processes at the epithelial cell level may critically contribute to the disease pathogenesis. The intestinal epithelium must interact with and adapt to the constant changing environment by processing the combined biological information from luminal enteric bacteria and host-derived signals [19]. IEC react on bacterial as well as immune-derived pro-inflammatory signals by secreting cytokines and chemokines like interleukin 6 (IL-6) and interferon γ-induced protein 10 (IP-10) to activate and attract Th1-immune cells and phagocytic cells to the site of infection. Cytokine and chemokine levels were shown to be strongly elevated in inflamed intestinal regions of IBD patients [20]. Experimental studies confirmed that IP-10 plays an outstanding role in uncontrolled disease development as the blockade of IP-10 by an anti-IP-10 antibody was sufficient to decrease disease severity in IL-10^−/−^ mice. This effect was shown to be due to reduced Th1 cell generation in inductive sites and reduced recruitment of Th1 effector cells to the colon [21]. Another experimental study revealed that anti-IP-10 therapy attenuates murine acquired immunodeficiency syndrome (MAIDS) colitis through the blockade of Th1-cell trafficking and the reduction of IEC apoptosis [22]. Although the importance of IP-10 as a pro-inflammatory mediator in IBD has been clearly demonstrated, the molecular mechanisms underlying the regulation of IP-10 expression in IEC during disease development are not well understood. Interestingly, recent studies revealed that probiotics are able to regulate chemokine expression in IEC. Although *E. coli* strain Nissle 1917 and VSL#3 were shown to reduce TNF-or *Salmonella dublin*-induced IL-8 chemokine expression in IEC [23], [24], the molecular mechanisms of target-specific inhibition of IEC activation through probiotic bacteria under conditions of chronic intestinal inflammation are not understood. The aim of our study was to analyze the molecular mechanisms of strain-specific effects of VSL#3 on IP-10 production in activated IEC. Furthermore, the effect of VSL#3 on TNF-induced ileitis in heterozygous TNF^ΔARE^ mice and on colitis in IL-10^−/−^ mice was investigated in the context of IP-10 expression in IEC.

## Materials and Methods

### Cell culture

The small intestinal epithelial cell line Mode-K [25] (passage 10–25) as well as the human embryonic kidney epithelial cell line HEK293 [26] was grown in a humidified 5% CO2 atmosphere at 37°C to confluency in 6, 12 or 24 well tissue culture plates (Cell Star, Greiner bio-one, Frickenhausen, Germany). TLR2 deficient HEK293 cells were bought from InvivoGen (Cat.Nr.: 293-null) and HEK293 cells expressing TLR2 were also bought from InvivoGen (Cat.Nr.: 293-mtlr2). The Mode-K cell culture media was Dulbecco's modified Eagle's medium (DMEM) containing 10% fetal calf serum (FCS), 1,0% Glutamine and 0,8% antibiotic antimycotic (Invitrogen, Carlsbad, USA). Glutamine was omitted for the cultivation of HEK293 cells and Blasticidin was added for the cultivation of the stably transfected HEK293 cells. Cell culture media was changed prior to stimulation.

### Cell culture stimulation and bacterial treatment

Confluent Mode-K cell monolayers were stimulated with TNF (10 ng/ml) (R&D Systems Europe), brefeldin A (0,5 µM) (Calbiochem), lactacystin (22 mM) (Biomol), NH_4_Cl (20 mM) (Sigma), 3-methyladenine (5 mM) and/or VSL#3 bacteria (VSL#3 mixture or single strains, generous gift from Dr. DeSimone, L'Aquila, Italy) (moi 20) for 24 h if not otherwise indicated. Viability of cells after brefeldin A treatment (6h) (93% of viable cells, data not shown) was analysed by a WST assay (Roche). VSL#3 derived *L. casei* and *L. plantarum* 299v were grown anaerobically (Anaerogen, Oxoid) at 37°C in MRS Broth (Fluka, Heidelberg) containing 0,05% L-cysteine (Roth). *E. coli* strain Nissle 1917 (a generous gift from Dr. Sonnenborn, Ardeypharm GmbH, Herdecke, Germany) was grown aerobically in LB-media. Bacteria were centrifuged (4500 g, 10 min) and resuspended in DMEM. *L. casei* was heat-killed (30 min, 90°C), fixed (fL.c) (3 hours, 5% formaldehyde, 4°C) or lysed by lysozyme (Sigma, 50 mg/ml) in filter sterilized 10 mM Tris buffer (pH 8). Fixed bacteria were washed three times with sterile PBS before IEC stimulation. For cell surface treatment, bacteria were incubated with Phospholipase A (2 mg/ml) (Fluka), Trypsin (2 mg/ml) (Roth) or Proteinase K (50 µg/ml) (Roth) in filter-sterilized Tris buffer (50 mM Tris, 0,1 M NaCl, pH 8, 1h, 37°C) in a shaker.

### Western blot

Purified primary IEC or Mode-K cells were lysed in Laemmli buffer and 50 µg of protein were subjected to electrophoresis on 10% or 15% SDS-PAGE gels. Anti IP-10 (R&D Systems Europe, Arlington), anti-IκB, anti-phospho-RelA (Cell Signaling, Beverly, MA), anti-ubiquitine (Cell Signaling, Beverly, MA), anti-DsRed (clontech) and anti-ß-actin-antibody (ICN, Costa Mesa, CA) were used to detect immunoreactive IP-10, IκB, phospho-RelA, DsRed, ubiquitine and ß-actin, using an enhanced chemoluminescence light-detecting kit (GE, Arlington Heights, IL).

### ELISA

IP-10 and IL-6 concentrations were determined in IEC supernatants using the appropriate ELISA kits (R&D Systems Europe) according to the manufacturer's instructions.

### Chromatin Immunoprecipitation (ChIP)

Mode-K cells in 75 cm^2^ flasks were pre-incubated with *L. casei* for 1 h and stimulated with TNF (10 ng/ml) for 2 h. Cells were fixed by formaldehyde fixation (1%) and nuclear extraction as well as chromatin immunoprecipitation were performed using a ChIP-kit (Active Motif, Carlsbad, CA, USA) according to the manufacturer's instructions. Immunoprecipitation was carried out over night at 4°C with an anti-NFkappaB p65 antibody (Cell Signaling, Beverly, MA). DNA/protein/antibody-complexes were collected with salmon sperm saturated protein A/G agarose for 30 min and washed three times in high salt buffer followed by three more washes in no salt buffer. DNA was released from the immune complex by heating and subsequent proteinase K treatment. DNA was then extracted by phenol-chloroform and eluted in water. The input control for the PCR was DNA from total nuclear extract. PCR was performed with total DNA (input control, 1 µl) and immunoprecipitated DNA (1 µl) using the following IP-10 promoter-specific primers 5′-AACAGCTCACGCTTTG, 5′-GTCCTGATTGGCTGACT. The length of the amplified product was 186 bp. PCR products (10 µl) were subjected to electrophoresis on 2% agarose gels.

### Transfection

Mode-K cells (luciferase assay) or HEK293 cells (IP-10 overexpression assay) (50% confluent, 24-well plate) were transfected using FuGENE (Roche, Mannheim, Germany) according to the manufacturer's instructions (24 h). A mixture of 23 µl DMEM (Invitrogen), 1.5 µl FuGENE and 0.5 µg of the appropriate plasmid (pGL3-basic-IP10 (p-IP-10), pGL3-basic (ctr-vector), pIP-10-DsRed, pDsRed-Monomer-C1 (ctr-DsRed)) was added to every well and incubated for 6h.

The pGL3-basic-IP10 plasmid was created by cloning a murine IP-10 promoter region (nucleotides −269 to −28 relative to the start point of transcription) into the *XhoI* and *BglII* sites of the pGL3-basic vector (Promega, Madison, WI), placing the promoter directly upstream of the firefly luciferase coding sequence. Therefore, the IP-10 promoter region was amplified from Mode-K cell genomic DNA via *pfu* (QIAGEN, Hilden) PCR. The sense primer was 5′-GCG*CTCGAG*CTCAAACAGCTCAC-3′ (the italic portion indicates the *XhoI* restriciton site) and the reverse primer was 5′-GCG*AGATCT*TCGAGTGCCGGCTG-3′ (the italic portion indicates the *BglII* restriction site). After digestion and ligation of PCR product and vector, the recombinant plasmid was verified by DNA sequencing (QIAGEN Sequencing Service, Hilden). The PGL3-basic-vector was used as a transfection control.

The vector pIP-10-DsRed contains a cytomegalovirus (CMV) promoter sequence driving the expression of the chemokine IP-10, which is N-terminally fused to Red Fluorescent Protein (DsRed). A 0.3 kb cDNA of murine IP-10, covering the whole open reading frame, was amplified from the Mode-K cell cDNA pool by *pfu* (QIAGEN, Hilden) PCR using primers designed according to the IP-10 cDNA sequence (GenBank Accession No. NM_021274.1). The sense primer for IP-10-DsRed was 5′-GCG*CTCGAG*AT**ATG**AACCCAAGTGCTGCCG-3′ (the italic portion indicates the *XhoI* restriction site and bold letters indicate the start codon). The antisense primer was 5′-GCG*CCCGGG*AA**TTA**AGGAGCCCTTTTAGACCT-3′ (the italic portion indicates the *XmaI* site; stop codon in bold letters). The PCR product was digested and cloned into the *XhoI* and *XmaI* sites of pDsRed-Monomer-C1 (Clontech, Mountain View, CA). The recombinant plasmid was verified by DNA sequencing (QIAGEN Sequencing Service, Hilden). pDsRed-Monomer-C1 (constantly expressing dsRed) was used as a transfection control.

### Luciferase assay

Transfected Mode-K cells were stimulated with TNF or TNF and *L. casei* for 24 h. Cells were lysed in 30 µl lysis buffer (Promega) and cell debris were separated by high speed centrifugation. Supernatants (25 µl) were mixed with Reagent A (PJK, Kleinblittersdorf) and the firefly luminescence was measured at 550 nm. A volume of 100 µl of Reagent B (PJK, Kleinblittersdorf) was added and renilla luminescence was measured at 480 nm. Relative luciferase activity was calculated in percentages: ((firefly/renilla)_sample_/(firefly/renilla)/_control_)×100.

### RNA isolation, reverse transcription and real-time PCR

RNA from Mode-K cells or isolated primary IECs was extracted using Trizol Reagent (Invitrogen, Karlsruhe, Germany) according to the manufacturer's instructions. Extracted RNA was solved in 20 µl water containing 0.1% diethyl-pyrocarbonate. RNA concentration and purity (A_260_/A_280_ ratio) was determined by spectrophotometric analysis (ND-1000 spectrophotometer, NanoDrop Technologies, Willigton, USA). Reverse transcription was performed using 1 µg total RNA. Real-time PCR was performed using 1 µl cDNA in a Light CyclerTM system (Roche Diagnostics, Mannheim, Germany) as previously described [27]. Primer sequences were: TLR2; sense 5′-TGGGGGTAACATCGCT and reverse 5′-CATCTACGGGCAGTGG, IP-10; sense 5′-TCCCTCTCGCAAGGAC and reverse 5′-TTGGCTAAACGCTTTCAT; 18S; sense 5′-CGGCTACCACAT-CCAAGGAA and reverse 5′-GCTGGAATTACCGCGGCT. The amplified product was detected by the presence of a SYBR green fluorescent signal. Melting curve analysis was used to document amplicon specificity and crossing points (Cp) were determined. Relative induction of gene mRNA expression was calculated according to the 2^−ΔΔCp^ [28] method and normalized to the expression of 18S. Data were expressed as fold change against untreated cells or wildtype mice. Presence or absence of TLR2 transcripts in the transfected HEK293 cells was proven by running a 2% Agarose gel with amplicons (321 bp) generated by reverse transcription of mRNA isolated of the HEK293 cells followed by real-time PCR.

### T cell transmigration assay

Murine splenocytes were isolated from a fresh spleen (mouse genotype: C57Bl/6/N) and activated with 2,5 µg/ml Concavalin A (Sigma) in RPMI (+10%FCS) at 37°C and 5% CO2 for 16 hours. Cells were then centrifuged and about 1×10^8^ cells were resuspended in 1 ml RPMI/25 mM Hepes. The cell suspension was then given onto 5 ml NycoPrep 1,077 A (AXIS-SHIELD, Oslo via Progen) and centrifuged (600 g, 20 min). The activated T lymphoblasts which accumulate at the interphase between media and NycoPrep were taken and resuspended in 30 ml washing buffer (RPMI/5%FCS/25mM Hepes). The cells were washed three times (250 g, 10 min) and resuspended to a final cell number of 2×10^6^ cells/ml transmigration media (RPMI/5%FCS/25mM Hepes). The activated murine T lymphoblasts were seeded on top of transwell filters (5 µm pore size) at a concentration of 2×10^5^ cells/ml transmigration media. The lower chamber was filled with 500 µl of transmigration medium and 100 µl of pure cell culture media or cell culture media from stimulation experiments with Mode-K cells (control, TNF, TNF/fL.c) or TNF-conditioned media supplemented with either a neutralizing anti-IP-10 antibody (30 µg/ml) (R&D Systems) or a control goat-IgG (30 µg/ml) (Dianova). The assay was then incubated for 2 hours (37°C/5% CO_2_) and the number of transmigrated cells in the lower chamber was analyzed using a CellCounter (Axiovision). The assay was performed in triplicates and the number of transmigrated cells at three representative regions (roi) of each single triplicate was measured.

### Co-immunoprecipitation

Mode-K cells (6-well plates) were stimulated with TNF alone or TNF and *L. casei* for 6 h. Cells were lysed in 200 µl of 1× lysis buffer (Cell Signaling, Beverly, MA) supplemented with PMSF (1 mM). Cell debris were removed by centrifugation (1400 g, 10 min) and supernatants were incubated with anti-IP-10-antibodies (R&D Systems Europe) (3h, 4°C) in a shaker. Samples were then incubated over night at 4°C in a shaker together with 13 µl of protein A/G beads (Santa, Cruz, Europe), which were previously washed twice with 1xlysis buffer (Cell Signaling, Beverly, MA). Beads were collected by centrifugation (5 min, 8000 g), washed twice with 1xlysis buffer and resuspended in 50 µl Laemmli buffer for subsequent Western blot analysis.

### Pulse-chase experiment

Mode-K cells (6-well plates) in DMEM (0,5% FCS) were stimulated with TNF during the pulse period (S^35^ Methionine/Cysteine Labeling Mix, 25 µCi/ml (Perkin Elmer)) (3h). After the pulse period, the cells were washed three times with 1×PBS and either lysed in 200 µl of 1xlysis buffer (Cell Signaling, Beverly, MA) supplemented with PMSF (1 mM) or they underwent a chase period of 3h with or without stimulation with *L. casei*. After the chase period, cells were washed three times with 1×PBS and lysed. Co-immunoprecipitation for IP-10 was performed as described and beads were resuspended in 20 µl of Laemmli buffer for subsequent electrophoresis on a 15% SDS gel. After drying the gel it was placed on a Kodak Storage Phosphor screen (Amersham bioscience) in a cassette (Amersham Bioscience) over night. The Storage Phosphor screen was then scanned by a Typhoon TRIO+ scanner (Amersham, Bioscience).

### Animals

Conventionally raised TNF^ΔARE^ mice (a generous gift from Kollias G., Institute for Immunology, Biomedical Sciences Research Center “Al. Fleming”, Greece) on C57BL/6 background and SPF-raised IL-10^−/−^ mice on 129 SvEv background as well as wildtype C57BL/6 and 129 SvEv mice were fed 1,3×10^9^ cfu of VSL#3 bacteria (a generous gift from Dr. DeSimone, VSL#3 pharma, Italy) in 13,2% (w/v) gelatine, 20% (w/v) glucose in water every weekday for 15 and 21 weeks starting at the age of three weeks. Placebo fed mice were used as controls, respectively. The gelatine was prepared freshly every third day. Mice were killed at the age of 18 and 24 weeks followed by sampling of gut content and IEC isolation. Sections of the distal ileum, cecal tip and distal colon were fixed in 10% neutral buffered formalin (Sigma Aldrich). Fixed tissues were hematoxylin-and eosin-stained and embedded in paraffin. Histology scoring was performed by blindly assessing the degree of lamina propria mononuclear cell infiltration, crypt hyperplasia, goblet cell depletion and architectural distortion, resulting in a score from 0 (not inflamed) to 12 (inflamed), as previously described [29]. Animal use was approved by the institution in charge (approval no. 55.2-1-S4-2531-74-06 and 32-2347/4+63).

### Isolation of primary mouse intestinal epithelial cells

Primary IEC were purified as previously described [27]. Briefly, either ileal or cecal and colonic tissue was cut into pieces and incubated (37°C, 15 min) in Mode-K cell culture media supplemented with 1 mM dithiothreitol (Roth, Karlsruhe, Germany). The tissue/IEC suspensions were filtered, centrifuged (7 min, 300 g, RT) and cell pellets were resuspended in DMEM containing 5% fetal calf serum. The remaining tissue was incubated in 30 ml PBS (10 min, 37°C) containing 1.5 mM EDTA (Roth, Karlsruhe, Germany). After filtration, the tissue was discarded and the cell suspension from this step was centrifuged as above. Finally, primary IEC were purified by centrifugation through a 20%/40% discontinuous Percoll gradient (GE Healthcare, Uppsala, Sweden) at 600 g for 30 min. Primary IEC were collected in lysis buffer for subsequent Western blot analysis or in trizol for subsequent RNA isolation. Purity of IEC was confirmed by anti-CD3+-Western blot analysis.

### DNA isolation from gut content and bacteria-specific PCR

Gut content DNA was extracted from 200 mg of content using the QIAamp DNA Stool Mini Kit (Qiagen) according to the manfacturer's instructions. *Streptococcus thermophilus (S. thermophilus)* specific PCR of the 16S–23S rRNA gene spacer region was performed as described previously [30].

### Immunohistochemical labeling

The cecum was dissected and immediately immersed in Tissue Tek OCT compound (Agar Scientific) and frozen in liquid nitrogen. Samples were stored under liquid nitrogen until analysed. 8 µm sections were cut using a Leica CM1950 cryostat, picked up onto polylysine coated slides and air dried at room temperature for 45 minutes. Sections were fixed in 4%paraformaldehyde 0.1M phosphate buffer (pH7.4) for 5 minutes before being washed with 6 changes of PBS (pH 7.4) over 20 minutes. Slides were then incubated with 0.1% Triton×100 (Sigma) in PBS for 3 minutes before being washed as above. Fc receptors were blocked (Fcγ III/II receptor from BD Pharminogen) and slides incubated in 10% BSA 5% normal donkey serum in PBS for 2 hrs at room temperature. Blocking buffer was removed by capillary action and the slides incubated over night at 4°C in either IP-10 goat polyclonal IgG (Santa Cruz G-15 sc-14641) or control goat IgG (Santa Cruz sc-2028) in 2% BSA. The slides were washed with 6 changes of PBS over 1 hour. Alexa Fluor 488 donkey anti goat IgG (Invitrogen Molecular Probes) was applied to the sections and incubated for 30 minutes at room temperature. The slides were washed as above, counter stained with DAPI and mounted in vectashield (Vector laboratories). Sections were viewed on a Zeiss Axioskop microscope using a FITC and DAPI filter set and imaged using an QIMAGING camera.

### Statistical analysis

Data were expressed as mean of triplicates +/− standard deviation. Statistical tests were performed using two-tailed Student test for the *in vitro* experiments. The *in vivo* feeding studies were analysed using rank sum test. Differences were considered significant if values were <0.05 (*) or <0.01 (**).

## Results

### VSL#3-derived *L. casei* specifically inhibits TNF-induced secretion of chemotactically functional IP-10

**Figure 1:**
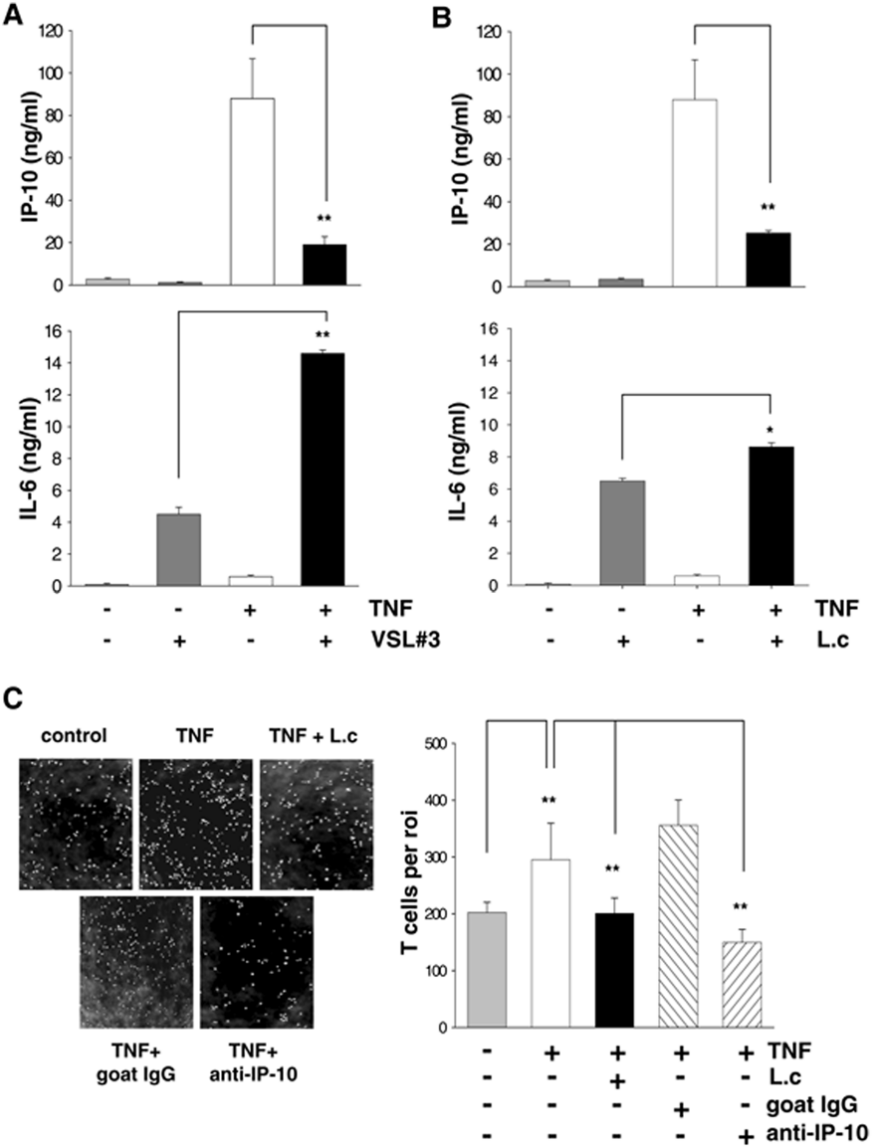
*L. casei* selectively inhibits TNF-induced secretion of chemotactically functional IP-10. Mode-K cells were stimulated for 24 h with (A) VSL#3 (moi 20) or (B) *L. casei* (moi 20) with or without TNF (10 ng/ml) activation for 24 h. The concentration of IP-10 and IL-6 in cell culture supernatants was measured by ELISA. Bars represent mean values (+/− SD) of triplicate samples. (C) 100 µl DMEM or cell culture supernatant from a 24 h stimulation experiment with Mode-K cells (control, TNF (10 ng/ml), TNF+*L. casei* (moi 20)) were added to 500 µl of transmigration medium. The amount of transmigrated cells per region of interest was determined by axiovision cell counting. Pictures show representative sections of transmigrated cells of three independent experiments and bars represent mean values (+/− SD) of triplicate samples.

To investigate the effects of VSL#3 on pro-inflammatory IP-10 expression in IEC, we stimulated Mode-K cells with VSL#3 bacteria (Moi 20) in the presence or absence of TNF, a potent inducer of IP-10 expression. The concentration of secreted IP-10 in the cell culture supernatant was analyzed by ELISA. VSL#3 did not induce IP-10 expression. In contrast, VSL#3 significantly reduced TNF-induced IP-10 but not IL-6 expression (Figure 1A, suggesting an IP-10 specific mechanism for the inhibitory function of IP-10. Besides, the stimulation of IEC with VSL#3 bacteria alone induced IL-6 expression. A series of additional experiments with the eight VSL#3 single bacterial strains (Moi 20) revealed that one of the eight strains, *L. casei*, exhibits analogous effects on IP-10 and IL-6 expression as the complex mixture VSL#3 (Figure 1B). *L. casei* was therefore identified as the effective bacterial strain in the probiotic mixture concerning the observed cytokine expression profile.

To analyze whether the observed inhibition of IP-10 has any functional consequence, we performed a T cell transmigration assay with Mode-K culture supernatants after a 24h stimulation experiment. Figure 1C shows that TNF-conditioned media increases the transmigration of activated T cells whereas supernatants of IEC that were costimulated with *L. casei* did not exert this chemotactic effect. To show that the reduced transmigration rate in the TNF/*L. casei*–conditioned media is due to the inhibition of IP-10 secretion by the probiotic bacteria, we used an IP-10 neutralizing antibody in the TNF-conditioned media. We demonstrated that the neutralization of IP-10 in this supernatant was sufficient to reduce the transmigration of T cells analogous to the effect of *L. casei*. These results clearly support the functional importance of the observed inhibition of IP-10 secretion in IEC by *L. casei.*

### Inhibition of TNF-induced IP-10 expression in IEC is mediated by a cell surface protein of *L. casei*

**Figure 2:**
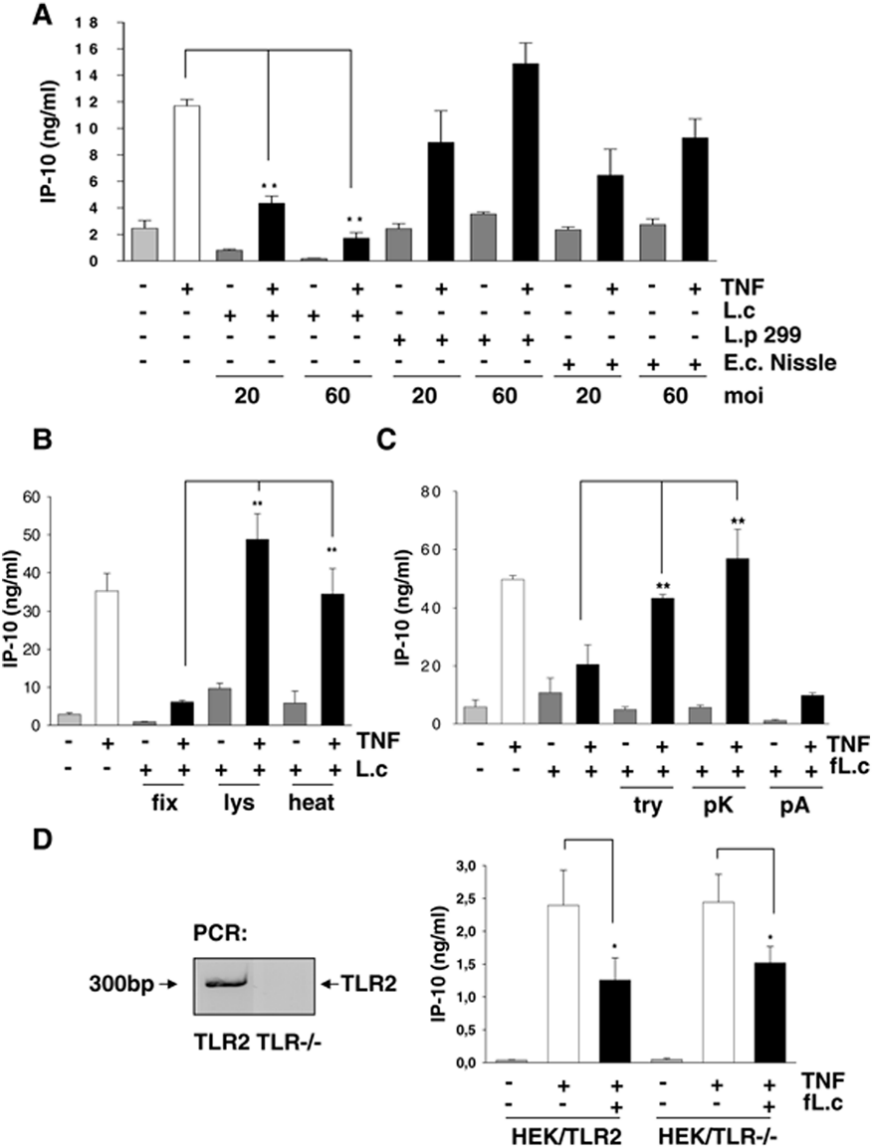
The inhibition of TNF-induced IP-10 secretion is mediated by a surface protein of *L. casei.* (A) Mode-K cells were stimulated for 24 h with *L. casei*, *Lactobacillus plantarum* 299v or *E. coli* Nissle 1917 (moi as indicated) with or without TNF (10 ng/ml) activation for 24 h. The concentration of IP-10 and IL-6 in cell culture supernatants was measured by ELISA. Bars represent mean values (+/− SD) of triplicate samples. (B) Mode-K cells were stimulated with *L. casei* (moi 20) killed by formaldehyde fixation (fix), lysozyme (lys)-digestion or heat (heat)-treatment with or without TNF (10 ng/ml) activation for 24 h. (C) *L. casei* was treated with trypsin (try), proteinase K (pK) or phospholipase A (pA) followed by formaldehyde-fixation (fL.c). (D) TLR2 deficient as well as TLR2 expressing HEK293 cells (absence/presence of TLR2 in the cells was analysed by real-time PCR) were stimulated for 24 h with TNF (10 ng/ml) and *L. casei* (moi 20). IP-10 concentration was measured in the culture supernatants by ELISA. The bars represent mean values (+/− SD) of triplicate samples.

Further analysis of other clinically relevant probiotic strains, *Lactobacillus plantarum* 299v and *E. coli* Nissle 1917, revealed that the inhibition of TNF-induced IP-10 secretion is dose-dependent and a unique feature of *L. casei* (Figure 2A). To investigate whether the inhibition of TNF-induced IP-10 expression was due to an active interaction between live *L. casei* and IEC, we inactivated *L. casei* by formaldehyde fixation, lysozyme lysis or heat treatment and subsequently stimulated Mode-K cells. Fig. 2B shows that fixed bacteria significantly inhibited TNF-induced IP-10 expression. However, lysis as well as heat-inactivation of *L. casei* resulted in complete loss of the observed inhibitory effect, suggesting a heat-labile surface structure of *L. casei* as the active bacterial component. To characterize this component in more detail, we treated *L. casei* with phospholipase A or the peptidases trypsine or proteinase K. Subsequent bacterial stimulation of Mode-K cells revealed that both peptidases completely reversed the effect of *L. casei* on TNF-induced IP-10 expression. In contrast, phospholipase A treatment did not affect the inhibitory effects of *L. casei* (Figure 2C). These results demonstrated that the inhibition of TNF-activated IP-10 expression by *L. casei* is likely mediated through a cell surface protein. Consistently, the inhibition of TNF-induced IP-10 expression was not dependent on the pattern recognition receptor TLR2 (Figure 2D). Of note, the inhibition of IP-10 was dependent upon the presence of *L. casei* during TNF stimulation, since the preincubation of IEC with *L. casei* did not result in an inhibition of subsequently TNF-induced IP-10 expression (data not shown).

### *L. casei* induces post-transcriptional inhibition of TNF-induced IP-10 expression in IEC

**Figure 3:**
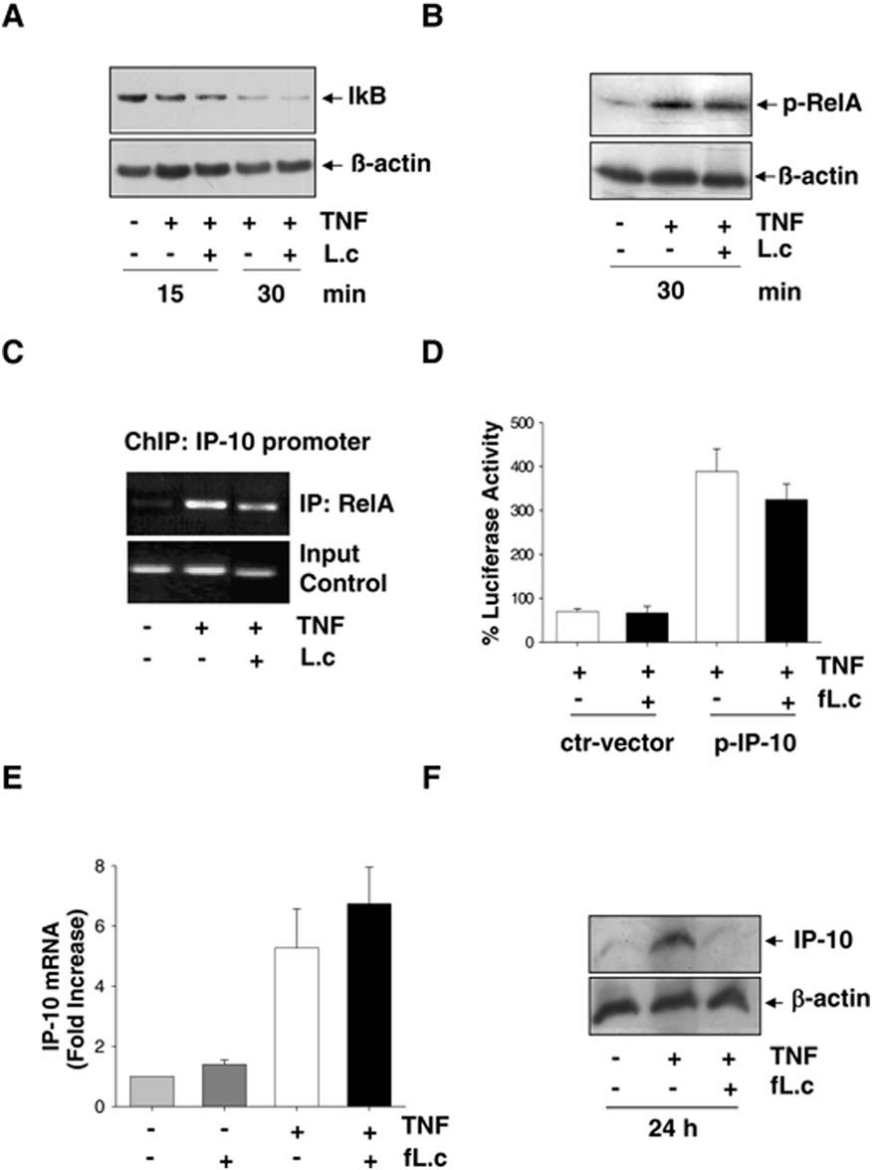
*L. casei*-induced inhibition of IP-10 secretion is mediated by a post-transcriptional mechanism (A) Mode-K cells were stimulated with TNF (10 ng/ml) and *L. casei* (moi 20) for 15 and 30 min and TNF-induced proteasomal degradation of IκB and (B) TNF-induced activation of RelA was analyzed by Western Blot in two independent experiments. (C) TNF-induced recruitment of NFκB RelA to the IP-10 promoter was analyzed in a ChIP experiment using an anti-RelA antibody after pre-incubation of Mode-K cells with *L. casei* (moi 20) (1h) and subsequent stimulation with TNF (10 ng/ml) for 2 h. (D) Control (ctr-vector)- and IP-10 reporter (p-IP-10)-plasmid transfected Mode-K cells were stimulated with TNF (10 ng/ml) with or without *L. casei* (moi 20) and intracellular luciferase activity was measured by a luminescence assay after 24 h. (E) Mode-K cells were stimulated with TNF (10 ng/ml) and/or *L. casei* (Moi 20) for 6 h followed by mRNA isolation, RT-PCR and qPCR. (F) Mode-K cells were stimulated with TNF (10 ng/ml) alone and together with *L. casei* (moi 20) for 24 h. Intracellular accumulation of IP-10 was analyzed by Western blot and the shown figure is representative for more than three independent experiments.

We were next interested in the molecular mechanism underlying the observed inhibitory effect of *L. casei* on IP-10 expression. Since the transcription factor NFκB plays an important role in the induction of IP-10 expression [31], we investigated whether NFκB signaling might be impaired by stimulation of IEC with *L. casei.* As shown in Figure 3A, TNF-induced IκB degradation as well as TNF-induced RelA phosphorylation (Figure 3B) was not inhibited by *L. casei*. In addition, ChIP analysis revealed that TNF-induced recruitment of RelA to the IP-10 promoter was not affected by the probiotic bacteria (Figure 3C). To investigate whether the activation of any other relevant transcription factor was blocked by *L. casei,* we transfected Mode-K cells with an IP-10-promoter reporter gene construct and observed that TNF-induced IP-10 promoter-dependent luciferase expression was not inhibited by *L. casei* (Figure 3D). Consistent with unaffected IP-10 promoter activity, TNF-induced increase of IP-10 mRNA levels was not inhibited by *L. casei* (Figure 3E). In contrast to the elevated IP10 transcript level, Western blot analysis revealed that intracellular IP-10 protein was almost completely lost after 24 hours of costimulation with *L. casei* (Figure 3F).

### Inhibition of IP-10 secretion is independent of the signal-specific activation of IEC

**Figure 4:**
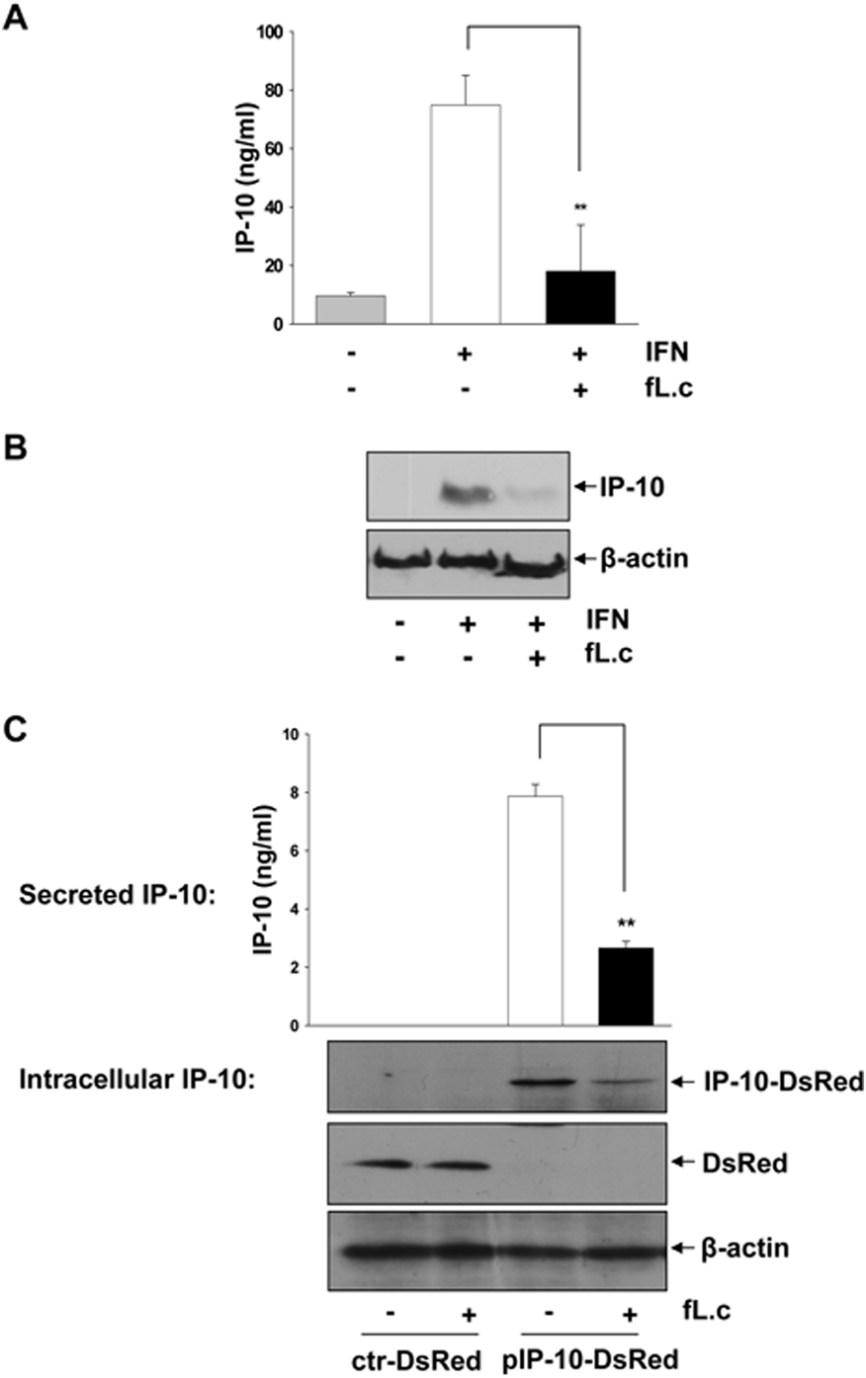
The inhibition of IP-10 secretion is independent of the signal-specific activation of IEC. (A) Mode-K cells were stimulated for 24 h with IFNγ (100 ng/ml) and *L. casei* (moi 20). Cell culture supernatants were analyzed by ELISA and (B) intracellular IP-10 protein levels by Western blot. (C) Mode-K cells were transfected with an IP-10 overexpression (pIP-10-DsRed)- or a ctr-DsRed-plasmid (transfection control) for 6 h and were then treated with *L. casei* for 24 h. IP-10 levels in the supernatants were determined by ELISA and intracellular DsRed-tagged IP-10 (IP-10-DsRed) or DsRed levels (transfection control) were determined by Western blot. Bars in A/C represent mean values (+/− SD) of triplicate samples and all figures are representative for three independent experiments.

We next performed a stimulation experiment with IFNγ, another potent inducer of IP-10 expression. As shown in Figure 4, IFNγ-induced IP-10 secretion (Figure 4A, ELISA analysis) and intracellular IP-10 protein expression (Figure 4B, Western blot analysis) was completely inhibited by *L. casei.* These results were analogous to the above described results observed under TNF-stimulation, suggesting that the inhibitory effects of *L. casei* were TNF-independent. To further characterize the signal-specificity of the inhibitory effect, we next performed a stimulation experiment with HEK293 cells that were transfected with a murine IP-10 overexpression plasmid resulting in constitutive expression of the chemokine. As shown in Figure 4C, *L. casei* significantly reduced intracellular and secreted levels of IP-10 in this experimental setup, whereas the production of the Ds-Red-control protein was not affected by the probiotic treatment. This result showed that the inhibitory effect of *L. casei* is indeed independent of the signal-specific activation pathways for IP-10, supporting our finding that the observed inhibition is mediated by a post-transcriptional mechanism.

### *L. casei* inhibits IP-10 secretion and triggers subsequent intracellular degradation of the chemokine

**Figure 5:**
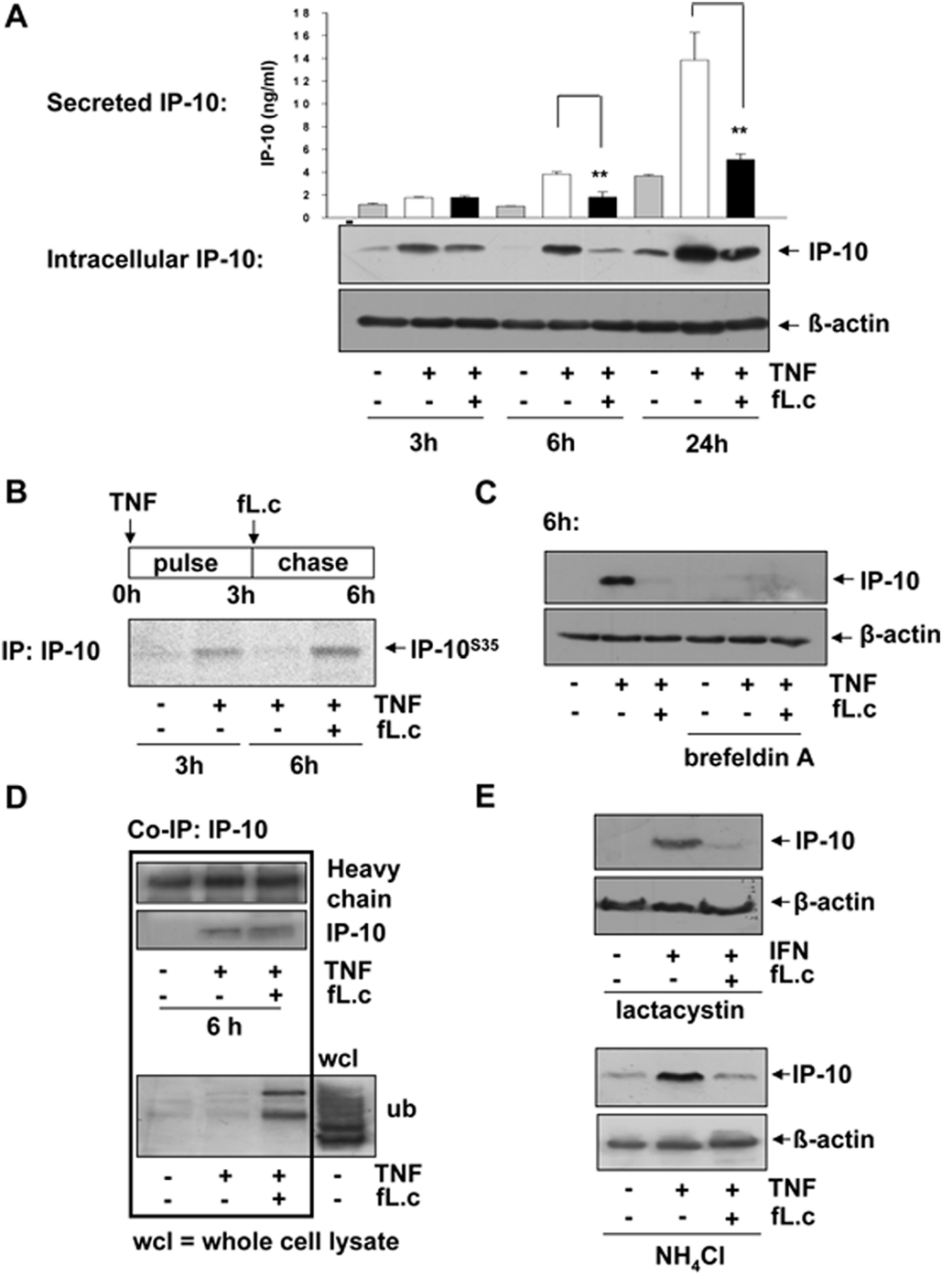
Degradation of IP-10 protein is the result of an IP-10 specific secretional blockade. (A) Mode-K cells were stimulated for 1, 3 or 24 h with TNF (10 ng/ml) alone or together with *L. case*i (moi 20) and intracellular as well as secreted levels of IP-10 were analyzed by Western Blot or ELISA analysis. Bars in A represent mean values (+/− SD) of triplicate samples. The shown figure is representative for three independent experiments. (B) Mode-K cells were stimulated with TNF (10 ng/ml) during a 3h pulse period (S^35^-labelled cysteine/methionine, 25 µCi) followed by a 3 h chase period in DMEM supplemented with *L. casei* (moi 20) or not. Subsequent immunoprecipitation for IP-10 was followed by protein separation on a 15% SDS gel. Intracellular levels of S^35^-labelled IP-10 were then made visible by a Phosphoimager plate. The shown figure is representative for two independent experiments. (C) Mode-K cells were stimulated with TNF (10 ng/ml) alone or together with *L. casei* in the presence or absence of brefeldin A (0,5 µM). The amount of intracellular IP-10 after 6 h of stimulation was analyzed by Western blot. (D) Mode-K cells were stimulated for 6 h with TNF (10 ng/ml) alone or with *L. casei* (moi 20) before lysis and subsequent immunoprecipitation using an anti-IP-10 antibody was performed. Western blot was performed to investigate the presence of IP-10 and ubiquitine in the precipitated fractions (single experiment). (E) Mode-K cells were stimulated with IFNγ (100 ng/ml) or TNF (10 ng/ml) alone or together with *L. casei* (moi 20) in the presence of lactacystin or NH_4_Cl for 24h and intracellular IP-10 was analyzed by Western blot. The shown figures are representative for three independent experiments.

We next investigated whether *L. casei* had an inhibitory effect on IP-10 protein translation. As shown in Figure 1, general protein translation and secretion was not impaired by the probiotic bacteria as indicated by normal or even increased secretion of other cytokines/chemokines like IL-6 (Figure 1A/B) or MIP-2 (data not shown). Western blot analysis of intracellular IP-10 protein levels revealed that TNF-induced IP-10 protein expression was not inhibited by *L. casei* at early time points (3h after costimulation). In contrast, the loss of intracellular IP-10 protein was detectable after 6 to 24 h of costimulation although it was not secreted into the cell culture supernatant (Figure 5A). This result demonstrated that initial TNF-induced IP-10 translation is not targeted by the probiotic bacteria suggesting that the observed loss of IP-10 protein is due to post-translational degradation. To further analyse the fate of initially produced IP-10 protein in more detail, we performed a S^35^ pulse-chase labelling experiment. Interestingly, IP-10 immunoprecipitation experiments showed that TNF-induced S^35^-IP-10 protein, that was produced during the initial 3h pulse period, was absent in Mode-K cells after the subsequent 3h chase period, demonstrating the functionality of the secretion system. In contrast, TNF-induced S^35^-IP-10 was still detectable intracellularly after the chase period in Mode-K cells that were costimulated during the chase period (Figure 5B). This result clearly showed that *L. casei* prevents the secretion of IP-10 protein. However, this finding was in sharp contrast to the observation that IP-10 protein is lost intracellularly at later time points instead of intracellular accumulation as it would be expected to be the result of a secretional blockade. To further analyze this discrepancy, we performed TNF and *L. casei* c(o)stimulation experiments in the presence of brefeldin A, a known inhibitor of vesicular transport from the endoplasmic reticulum to the golgi network. Surprisingly, we found that the inhibition of the protein export machinery by brefeldin A resulted in complete loss of intracellular IP-10 protein analogous to the results described above in the costimulation experiments with *L. casei* (Figure 5C). This result confirms that indeed, a secretional blockade of IP-10 results in subsequent loss of intracellular IP-10 protein, suggesting the initiation of degradative mechanisms as a consequence of initial IP-10 accumulation.

We next addressed the question which degradation pathway might be responsible for the observed loss of IP-10 protein in IEC. Co-immunoprecipitation analysis revealed that IP-10 was ubiquitinated in the presence of *L. casei* (Figure 5D) indicating proteasomal degradation. Since TNF-induced IP-10 expression depends on proteasomal IκB degradation, we stimulated cells with IFNγ to investigate the effect of proteasomal inhibition on IP-10 degradation. Figure 5E shows that stimulation with the proteasomal inhibitor lactacystin did not rescue IFNγ-induced IP-10 protein from degradation. Next, we investigated whether IP-10 protein was degraded through lysosomal pathways. The inhibition of lysosomal degradation by NH_4_Cl (Figure 5E) did not prevent the loss of IP-10 protein, suggesting an additional proteolytic mechanism for IP-10 degradation.

### *L. casei* mimicks the effects of the inhibition of autophagic-lysosomal pathways on cytokine/chemokine expression

**Figure 6:**
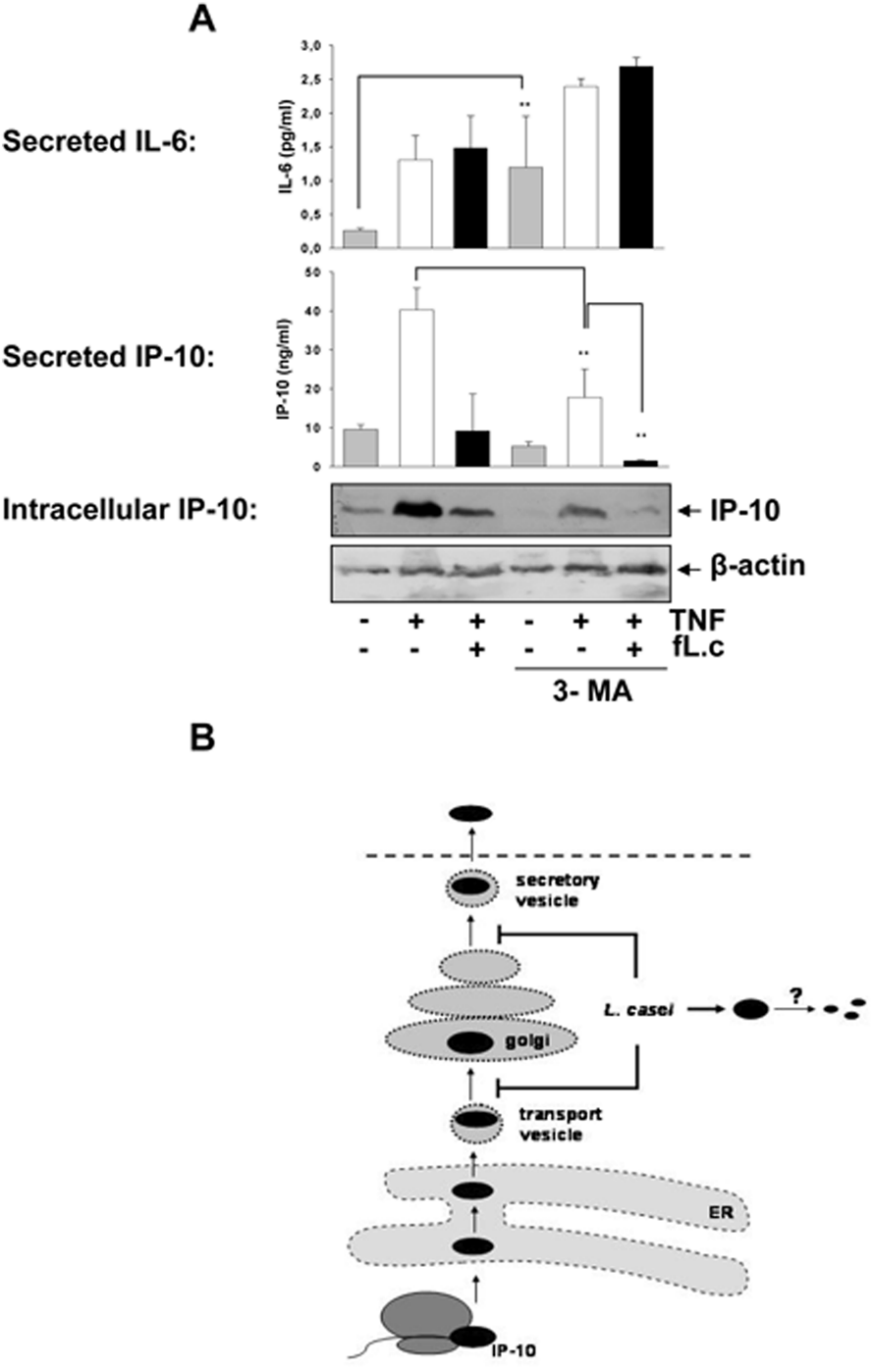
Blockade of 3-MA-dependent vesicular transport by *L. casei* might induce the degradation of IP-10. (A) Mode-K cells were stimulated with TNF (10 ng/ml) alone or together with *L. casei* (moi 20) in the presence or absence of 3-MA for 24 h and the amount of IP-10 in the cell culture supernatant as well as intracellularly was analyzed by ELISA and Western blot. Figure A is representative for three independent experiments and bars represent mean values (+/− SD) of triplicate samples. (B) The scheme illustrates our hypothesis that *L. casei* might target 3-MA dependent vesicular transport of IP-10 resulting in a secretional blockade and subsequent degradation of the chemokine.

To investigate whether autophagic-lysosomal degradation pathways might play a role in the observed loss of IP-10 protein, we applied TNF and *L. casei* to the epithelial cell culture in the presence of 3-methyladenine (3-MA). Surprisingly, we demonstrated that the stimulation of IEC with 3-MA, which is a known inhibitor of vesicle formation in the context of autophagy-lysosomal degradation, did not prevent the loss of intracellular IP-10 (Figure 6A, Western blot analysis), but rather mimicked the effects of *L. casei* on the TNF-induced cytokine secretion pattern (Figure 6A, ELISA analysis). Figure 6A shows that 3-MA decreased the level of IP-10 protein intracellularly as well as in the cell culture supernatant. In contrast, TNF-induced IL-6 secretion was not inhibited in the presence of 3-MA. These observations indicated that IP-10 but not IL-6 secretion is dependent on the formation of secretory vesicles that can be inhibited by 3-MA. We therefore hypothesize that *L. casei* impairs 3-MA-dependent vesicular trafficking resulting in disturbed IP-10 secretion and subsequent degradation of IP-10 protein (Figure 6B).

### The protective activity of VSL#3 on experimental colitis is intestinal segment specific and correlates with IP-10 expression in IEC

**Figure 7:**
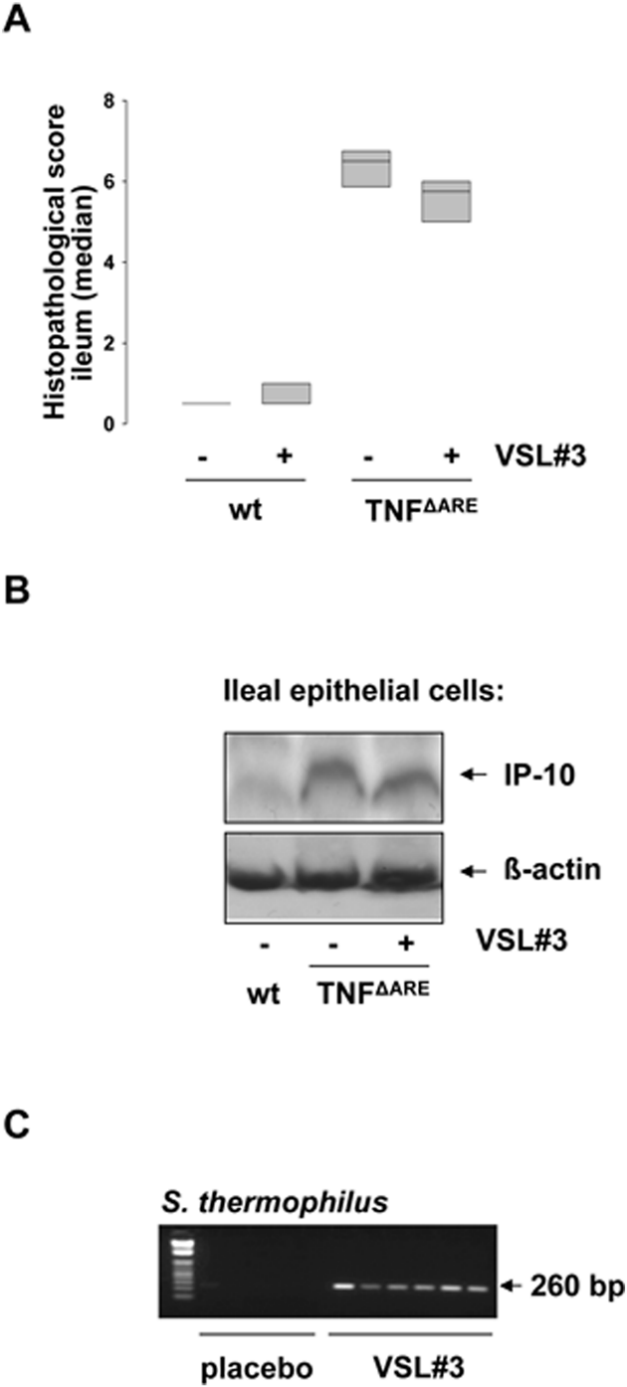
VSL#3 does not reduce experimental ileitis in TNF^ΔARE^ mice. TNF^ΔARE^ (n = 6) and wt mice (n = 5) were fed VSL#3 (1,3×10^9^ cfu/mouse/day) or placebo for 15 weeks. Mice were sacrificed at 18 weeks of age and histopathological scoring was performed by blindly assessing the inflammation between 0 (not inflamed) and 12 (highly inflamed). Figure A shows the median histopathologic score of TNF^ΔARE^ mice in the study. (B) Ileal IEC were isolated and pooled proteins (50 µg) of all mice in a group were analyzed for IP-10 expression by Western blot. (C) The presence of VSL#3 derived *S. thermophilus* (S.t) in the gut of the TNF^ΔARE^ mice was examined by bacteria-specific PCR analysis.

**Figure 8:**
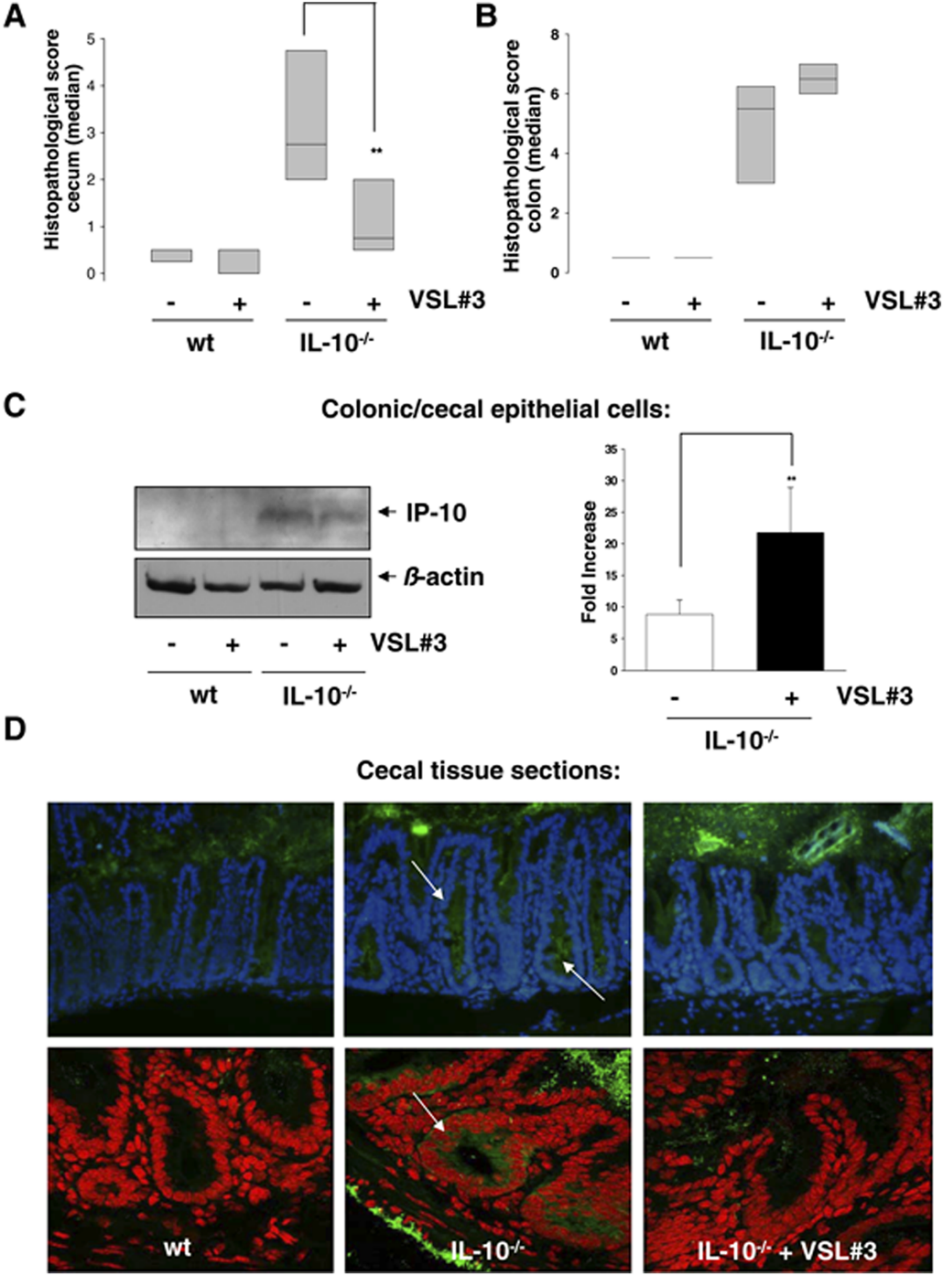
Intestinal segment specific effects of VSL#3 on experimental inflammation correlate with IP-10 expression in IEC. IL-10^−/−^ mice were fed VSL#3 (1,3×10^9^ cfu/mouse/day) (n = 14) or placebo (n = 12) analogous to wt mice (n = 9) for 21 weeks. Mice were sacrificed at 24 weeks of age and histopathological scoring was performed by blindly assessing the inflammation between 0 (not inflamed) and 12 (highly inflamed). Figure A and B show the median histopathologic score of the cecum and colon of IL-10^−/−^ mice in the study. (C) Cecal and colonic IEC were isolated together and proteins (50 µg) as well as mRNA of all mice in a group were analyzed for IP-10 expression by Western blot or RT-PCR (D) Immunohistochemical staining of IP-10 in cecal tissue sections was performed. The upper row shows representative pictures (three mice per group were analysed) of stained tissue sections (IP-10 (green), DAPI (blue)) and the lower row shows representative false colour confocal images of the stained tissue sections ((IP-10) green, DAPI (red)). Arrows indicate regions that show elevated IP-10 expression.

To evaluate the physiological impact of VSL#3 on experimental intestinal inflammation, we performed probiotic feeding studies with heterozygous TNF^ΔARE^ mice, an animal model for TNF-induced experimental ileitis [32], and IL-10^−/−^ mice, an animal model for experimental colitis [33]. TNF^ΔARE^ mice, IL-10^−/−^ mice and wildtype mice were fed VSL#3 (1.3×10^9^ cfu per mouse/day) for 15 and 21 weeks post-weaning. Histopathological analysis of distal ileal sections revealed that VSL#3 did not inhibit the development of severe ileal inflammation in TNF^ΔARE^ mice (Figure 7A). As expected, we found that TNF-induced ileitis correlates with enhanced IP-10 protein expression in primary ileal epithelial cells from TNF^ΔARE^ mice. The absence of T-cell contaminations in the purified IEC from inflamed mice confirmed the purity of the epithelial cell isolation (data not shown). Consistent with the tissue pathology, VSL#3 did not reduce IP-10 protein expression in primary ileal IEC (Figure 7B). The presence of VSL#3 bacteria in the intestine was proven by the use of bacteria-specific PCR on DNA isolated from intestinal contents (Figure 7C). In contrast to the unresponsiveness of heterozygous TNF^ΔARE^ mice to VSL#3 treatment, we found that cecal (Figure 8A) but not colonic (Figure 8B) inflammation in IL-10^−/−^ mice was significantly reduced by the probiotic mixture, suggesting intestinal segment specific effects of VSL#3. In consistence with the results derived from TNF^ΔARE^ mice, we detected elevated levels of IP-10 in isolated intestinal epithelial cells of IL-10^−/−^ mice compared to wildtype mice. Although we were not able to separate cecal and colonic epithelial cells during the isolation procedure (for the sake of yield), Western blot analysis showed that VSL#3 clearly reduced the expression of IP-10 even in the pooled cell population. Real-time PCR analysis of IP-10 mRNA levels in these primary IECs showed that the observed reduction of IP-10 protein is not due to reduced levels of IP-10 mRNA (Figure 8C), supporting our finding that the reduction of IP-10 protein in IEC is the result of a post-transcriptional mechanism induced by VSL#3. Most importantly, immunohistochemical staining of IP-10 in cecal tissue sections confirmed that VSL#3 strongly reduced IEC-specific IP-10 expression in the cecum of IL10^−/−^ mice (Figure 8D).

## Discussion

The present study shows for the first time bacterial strain-specific effects of the clinically relevant probiotic mixture VSL#3 on the expression of a pro-inflammatory chemokine in primary IEC and IEC lines. VSL#3-derived *L. casei* was identified to inhibit TNF-induced IP-10 expression to the same extent as the complex probiotic mixture. This effect was found to be a unique feature of VSL#3-derived *L. casei* as other clinically relevant probiotic bacteria like *L. plantarum* 299 and *E. coli* Nissle 1917 did not reduce IP-10 secretion. Since formaldehyde fixed *L. casei* had the same inhibitory effect on IP-10 secretion as live bacteria, active interaction between bacteria and IEC is not necessary for the inhibitory effect, suggesting a bacterial cell surface component as the effective probiotic structure. In contrast to *L. rhamnosus* GG, a probiotic bacteria that was shown to have protective effects on IEC survival and growth via two secreted proteins [34], we demonstrated that the inhibition of TNF-induced IP-10 secretion was due to a heat-labile surface protein of *L. casei.* The proteinasous nature of the bacterial structure responsible for the inhibition of IP-10 was supported by the finding that the effect was independent of TLR2-dependent signalling mechanisms. Stimulation experiments with IFNγ and IP-10 overexpression experiments revealed stimuli-independent mechanisms to underly the inhibition of IP-10 expression by *L. casei*. Given the high physiological relevance of elevated IP-10 expression in inflamed intestinal tissue regions of IBD patients and animal models of experimental colitis [20]–[22], the inhibitory effect on this chemokine may play an important role in the anti-inflammatory effects of the probiotic mixture VSL#3 on inflammatory bowel diseases [9]. We were able to show that IP-10 expression in IEC correlates with inflammation, as IP-10 expression was elevated in primary ileal IEC from heterozygous TNF^ΔARE^ mice as well as in colonic IEC from IL-10^−/−^ mice compared to healthy wildtype mice. In a healthy host, pathogen-induced secretion of IP-10 triggers enhanced recruitment of effector Th1 cells and monocytes into the mucosa, resulting in subsequent deletion of the infectious agent. In the case of IBD, chronically activated IEC steadily produce high amounts of IP-10 in the absence of any pathogenical stimuli resulting in constant Th1 and monocyte recruitment. The presence and activity of these effector cells may lead to the loss of epithelial cell integrity and tissue damage. In consequence, the expression of IP-10 in IEC has to be tightly regulated to prevent infections on the one hand and the development of pathological conditions like chronic intestinal inflammations on the other hand. Consistently, IP-10 expression has been shown to be modulated at various levels. The activation of PPARγ by PPARγ ligands inhibited IP-10 expression by reducing IP-10 promoter activity [35]. In addition, butyrate inhibited IFNγ and TNF-induced IP-10 transcription in colonic subepithelial myofibroblasts [36] and interleukin 10 suppressed LPS-induced IP-10 gene transcription in peritoneal macrophages [37]. Many studies have analyzed the transcriptional regulation of cytokines and chemokines like IP-10 but although post-transcriptional and post-translational regulation of chemokines is suggested to be very important [38], exact mechanisms are mostly unknown. The pro-inflammatory chemokine interleukin 8 was shown to be upregulated by a TLR5-mediated, p38-dependent post-transcriptional mechanism [39] and furthermore, to be processed at the amino terminal end by a matrix metallo proteinase [40]. In addition, the biosynthesis of IFNγ was shown to be suppressed post-transcriptionally by the stimulation with lead [41]. Concerning IP-10, complex post-transcriptional regulatory mechanisms seem to play an important role. Recent studies revealed regulatory mechanisms like IP-10 mRNA half-life modulation in monocytes [42] or C-terminal truncation through furin, a pro-protein convertase, resulting in another active form of the chemokine [43]. Furthermore, IP-10 was found to be processed C-terminally by gelatinase B and neutrophil collagenase [40]. In addition, the activity of IP-10 was shown to be regulated by its oligomerization status [44]. Our study revealed for the first time that probiotic bacteria are able to induce post-translational regulation of IP-10 protein in IEC. Decreased IP-10 protein stability was identified as a new post-translational regulatory mechanism.

*L. casei*-induced ubiquitination of IP-10 is very likely involved in the observed degradation of the chemokine although it does not target the protein for proteasomal degradation. It has already been shown that stimulation with *L. casei* has multiple effects on the expression of genes involved in proteasome and ubiquitination pathways in IEC [45], but the exact mechanisms are not known yet. In contrast to lysosomal degradation of LPS-induced TNF under hypoxia [46], lysosomal pathways were not involved in *L. casei*-induced loss of IP-10 protein, suggesting alternative degradation pathways to be involved. The inhibition of protein secretion through brefeldin A was found to induce degradation of IP-10 instead of intracellular accumulation, as it was shown for other secretory proteins like makrophage inflammatory protein-2 [47]. This result led to the hypothesis that *L. casei* might selectively impair secretion of IP-10 followed by subsequent degradation as a protective mechanism to prevent protein accumulation. In contrast to interleukin-6, the secretion of IP-10 was found to be dependent on tightly regulated vesicular transport. *L. casei* mimicks the effect of 3-methyladenine, an inhibitor of vesicular trafficking and exosome secretion [48], which specifically inhibits IP-10 secretion and induces loss of intracellular IP-10 protein. Considering the extensive post-translational processing of IP-10, this inhibitory mechanism might be due to the necessity for chemokine maturation processes in endo-lysosomal compartments prior to exocytosis as it has been shown for several other secretory proteins [49], [50]. We therefore hypothesize that *L. casei* inhibits vesicle trafficking similar to 3-methyladenine, resulting in impaired maturation and secretion of IP-10 followed by subsequent degradation of the chemokine (Figure 6B).

Our *in vivo* feeding studies revealed that VSL#3 has no effect on the development of ileal inflammation in heterozygous TNF^ΔARE^ mice. This result may indicate that the constant overexpression of TNF in these mice and the resulting T-cell driven immunopathology constitute such a strong pro-inflammatory stimulus that probiotic therapy is not sufficient to reduce tissue inflammation. Apart from that, one could raise the hypothesis that probiotic therapy is generally less effective in the ileum than in the colon due to high luminal content passage rates in this intestinal segment. The failure of VSL#3 in the context of ileitis together with the observed reduction of cecal inflammation in IL-10^−/−^ mice is consistent with clinical data showing effective probiotic treatment mostly in the context of UC [9] and pouchitis patients [51]. The finding that VSL#3 has protective effects on cecal but not on colonic inflammation clearly reveals intestinal segment specific effects of the probiotic mixture. The differences in response to probiotic treatment between the two intestinal compartments might be due to the lower inflammatory grade in the cecum compared to the colon in IL-10^−/−^ mice or due to differences in the bacterial colonisation. The discrepancy to previously published studies, showing anti-inflammatory effects of VSL#3 in the cecum and colon [15], may presumably be due to differences in the composition of the intestinal microbiota of the IL-10^−/−^ mice in different animal facilities or the time point of intervention. The intestinal microbiota has already been shown to have an enormous impact upon the onset, severity and localisation of experimental colitis, suggesting that even small differences might impact the effectiveness of probiotic treatment [52].

The expression of IP-10 in primary intestinal epithelial cells was found to correlate strongly with disease severity. Consistent with the finding that injection of an anti-IP-10 antibody leads to the attenuation of colitis in IL-10^−/−^ mice [21], our study revealed that the post-transcriptional inhibition of IP-10 secretion in cecal epithelial cells by VSL#3 is paralleled by reduced cecal inflammation. Our findings demonstrate that the inhibition of IP-10 secretion by VSL#3 is an important molecular mechanism contributing to the anti-inflammatory effect of the clinically relevant probiotic mixture.
