# Correction: Post-Translational Inhibition of IP-10 Secretion in IEC by Probiotic Bacteria: Impact on Chronic Inflammation

**DOI:** 10.1371/annotation/583d95a8-c18c-4c66-92be-1b1505802d86

**Published:** 2009-06-02

**Authors:** Gabriele Hoermannsperger, Thomas Clavel, Micha Hoffmann, Caroline Reiff, Denise Kelly, Gunnar Loh, Michael Blaut, Gabriele Hölzlwimmer, Melanie Laschinger, Dirk Haller

The spelling of the first author's last name is incorrect. The correct spelling is: Hoermannsperger. The corrected citation of this article is: Hoermannsperger G, Clavel T, Hoffmann M, Reiff C, Kelly D, et al. (2009) Post-Translational Inhibition of IP-10 Secretion in IEC by Probiotic Bacteria: Impact on Chronic Inflammation. PLoS ONE 4(2): e4365. doi:10.1371/journal.pone.0004365

